# Real-World Faricimab for Treatment-Naïve Neovascular AMD and Diabetic Macular Edema: 24-Month Outcomes from a Single-Center Pilot Cohort in South-Eastern Europe

**DOI:** 10.3390/medicina62071307

**Published:** 2026-07-06

**Authors:** Maja L. J. Živković, Marko Zlatanović, Nevena Zlatanović, Mladen Brzaković, Mihailo Jovanović

**Affiliations:** 1 Ophthalmology Clinic, University Clinical Center Niš, Bulevar dr Zorana Đinđića 48, 18000 Niš, Serbia; drzlatanovicmarko@gmail.com; 2 Department of Ophthalmology, Faculty of Medicine, University of Niš, Bulevar dr Zorana Đinđića 81, 18000 Niš, Serbia; 3 Community Health Center Niš, Vojvode Tankosića 15, 18000 Niš, Serbia; drnevenazlatanovic@gmail.com; 4 Special Hospital for Ophthalmology “Clinic Maja”, Vizantijski Bulevar 8, 18000 Niš, Serbia; brzi.92@hotmail.com; 5 Department of Ophthalmology, Faculty of Medicine, University of Kragujevac, Svetozara Markovića 69, 34000 Kragujevac, Serbia; drmihailojovanovic@gmail.com; 6 Ophthalmology Clinic, University Clinical Center Kragujevac, Zmaj Jovina 30, 34000 Kragujevac, Serbia

**Keywords:** faricimab, neovascular age-related macular degeneration, diabetic macular edema, intravitreal injection, angiopoietin-2, real-world outcomes, optical coherence tomography, bispecific antibody

## Abstract

*Background and Objectives:* Faricimab, the first bispecific antibody targeting VEGF-A and angiopoietin-2, has demonstrated durable efficacy in pivotal phase 3 trials for neovascular age-related macular degeneration (nAMD) and diabetic macular edema (DME). Real-world data on treatment-naïve patients managed with fixed-interval maintenance protocols, particularly from South-Eastern Europe, remain limited. This pilot study evaluated 24-month outcomes of intravitreal faricimab in treatment-naïve nAMD and DME, using a standardized four-injection loading phase followed by fixed every-16-week (Q16W) maintenance. *Materials and Methods:* This study conducted a retrospective, observational, single-center pilot cohort study of 20 consecutive treatment-naïve eyes (9 nAMD, 11 DME). All patients received four monthly loading injections followed by a fixed every-16-week (Q16W) maintenance schedule, supplemented by discretionary additional injections for residual or recurrent disease activity (215 injections total; mean 10.75 ± 0.79 per patient; range 9–12). Primary outcomes were changes in central foveal thickness (CFT) and best-corrected visual acuity (BCVA; Snellen lines with ETDRS letter equivalents) at months 4 and 24. Prespecified secondary analyses included bootstrap 95% confidence intervals, a linear mixed-effects model with a time × disease-group interaction, Bayesian credible intervals with weakly informative priors, false-discovery-rate (FDR) correction, and a minimum detectable effect-size analysis. *Results:* All 20 eyes completed 24-month follow-up. In nAMD, mean CFT decreased by 186.9 ± 71.9 µm (35.9%; bootstrap 95% CI 148.1–236.0; *p* < 0.001; d = 2.60), and BCVA improved by 3.89 ± 0.78 Snellen lines (~19 ETDRS letters; 95% CI 3.44–4.33; *p* < 0.001; d = 4.97). In DME, CFT decreased by 197.7 ± 65.7 µm (39.3%; 95% CI 162.5–237.3; *p* < 0.001; d = 3.01), and BCVA improved by 4.55 ± 1.04 lines (~23 ETDRS letters; 95% CI 4.00–5.09; *p* < 0.001; d = 4.39). All 20 eyes (100%) achieved ≥ 3 Snellen lines gain and ≥20% CFT reduction; 80% reached final BCVA ≥ 7 lines. A linear mixed-effects model showed a significant time effect (*p* < 0.001) but no time × group interaction (CFT *p* = 0.84; BCVA *p* = 0.51), indicating concordant trajectories across diseases. Bayesian analysis with weakly informative priors yielded posterior P(|d| > 0.8) ≥ 0.99 for all primary outcomes. After FDR correction, all pre-specified primary comparisons remained significant. The minimum detectable effect size with the realized sample sizes (Cohen’s d ≈ 0.66 combined, 1.07 nAMD, 0.94 DME at 80% power) was substantially below all observed effect sizes. No ocular or systemic adverse events were recorded. *Conclusions:* In this small, single-center, treatment-naïve pilot cohort, a fixed Q16W faricimab maintenance schedule with discretionary additional injections was associated with durable anatomical and functional improvements over 24 months in both nAMD and DME, with no adverse events recorded across 215 injections. Given the limited sample, these findings should be regarded as hypothesis-generating. The high responder rates likely reflect the cohort’s substantial baseline visual impairment (mean baseline BCVA ~20/120–20/200), which provides greater absolute capacity for measurable gain than in higher-acuity registration trial populations. These pilot data support fixed-interval faricimab as a logistically feasible candidate strategy in resource-constrained settings and should be confirmed in larger multicenter cohorts using standardized ETDRS acuity assessment.

## 1. Introduction

Neovascular age-related macular degeneration (nAMD) and diabetic macular edema (DME) are leading causes of irreversible central visual loss in adults in high-income countries [1,2]. The global burden of both conditions is substantial and rising: 196 million individuals were estimated to be affected by AMD in 2020, with projections reaching 288 million by 2040 [1]; the International Diabetes Federation projected 537 million adults living with diabetes mellitus globally in 2021 and 783 million by 2045, with the prevalence of clinically significant macular edema expected to rise from 18.83 million in 2020 to 28.61 million by 2045 [3]. Despite distinct pathophysiological origins—choroidal neovascularization in nAMD and hyperglycemia-driven blood–retinal barrier breakdown in DME—both conditions share the central mechanistic feature of vascular endothelial growth factor A (VEGF-A)-mediated macular exudation.

The pathophysiology of DME is distinct from that of nAMD and warrants specific consideration. Chronic hyperglycemia initiates a cascade of biochemical and microvascular injury, including pericyte loss, basement-membrane thickening, and capillary nonperfusion, that progressively compromises the inner blood–retinal barrier. The resulting endothelial dysfunction, together with leukostasis and a chronic, low-grade inflammatory milieu, promotes upregulation of vascular endothelial growth factor A (VEGF-A) and pro-inflammatory cytokines, increasing vascular permeability and driving the accumulation of intraretinal fluid that characterizes macular edema. Angiopoietin-2 contributes critically to this process: by antagonizing Tie2 signaling, it destabilizes the retinal vasculature and sensitizes the endothelium to VEGF-A, amplifying barrier breakdown. This convergence of VEGF-A- and Ang-2-mediated permeability provides the mechanistic rationale for simultaneous dual inhibition of both pathways in DME.

The introduction of intravitreal anti-VEGF therapy transformed the clinical management of both diseases. Ranibizumab in the MARINA and ANCHOR trials [4] and aflibercept in the VIEW 1/2 and VISTA/VIVID trials [5], together with comparative outcomes from the Diabetic Retinopathy Clinical Research Network Protocol T [6], established intravitreal anti-VEGF as the global standard of care. However, real-world outcomes of anti-VEGF monotherapy have consistently lagged behind trial efficacy, primarily due to undertreatment, suboptimal adherence, loss to follow-up, and the logistical burden of frequent dosing [7]. A key insight from the past decade is that VEGF-A inhibition alone is mechanistically insufficient. Angiopoietin-2 (Ang-2), a ligand of the endothelial Tie2 receptor and a critical regulator of vascular stability, has emerged as a complementary mediator: it destabilizes the choroidal vasculature in nAMD [8], drives disruption of the inner blood–retinal barrier in DME [9], and sustains exudation despite adequate VEGF-A blockade. This rationale led to the development of faricimab (Vabysmo; Genentech/Roche), the first bispecific antibody approved for intravitreal use, which independently neutralizes both VEGF-A and Ang-2 within a single molecular construct [10].

The pivotal phase 3 program comprised four trials. In nAMD, TENAYA and LUCERNE demonstrated that faricimab dosed up to every 16 weeks (Q16W) was non-inferior to aflibercept every 8 weeks (Q8W) for mean BCVA change over one year, with superior CST reductions in the matched-dosing phase [11]; two-year data confirmed sustained gains, with 59.0–66.9% of patients reaching Q16W dosing by week 112 [12]. In DME, YOSEMITE and RHINE showed that faricimab (Q8W or personalized treat-and-extend, T&E, up to Q16W) was non-inferior to aflibercept Q8W at one year, with greater CST reductions and higher fluid-resolution rates; at two years, over 60% of T&E patients had achieved Q16W dosing, with mean CST reductions of 197–216 µm [13].

Despite this evidence base, several gaps remain. First, most published real-world faricimab data come from switch or conversion cohorts, with limited long-term outcome data in *treatment-naïve* patients [14,15]. Second, the personalized T&E paradigm used in registration trials requires high-frequency monitoring and flexible scheduling, which may be infeasible in resource-constrained healthcare systems, raising the practical question of whether fixed-interval Q16W maintenance can deliver comparable results. Third, real-world evidence from South-Eastern Europe is almost entirely absent from the published literature.

The aim of this study was to characterize 24-month anatomical and functional outcomes of intravitreal faricimab in a focused, methodologically homogeneous, treatment-naïve cohort of consecutively enrolled patients with nAMD or DME, treated at a single tertiary ophthalmology center in Niš, Serbia, with a standardized four-injection loading phase followed by fixed Q16W maintenance. We hypothesized that fixed Q16W maintenance in a treatment-naïve population would yield durable improvements in CFT and BCVA consistent with the magnitude of effect reported in the pivotal phase 3 trials.

## 2. Materials and Methods

### 2.1. Study Design and Ethical Framework

This retrospective, single-center, observational cohort study evaluated 24-month anatomical and functional outcomes of intravitreal faricimab in treatment-naïve patients with neovascular age-related macular degeneration (nAMD) or diabetic macular edema (DME). All participants were managed at the Special Hospital for Ophthalmology “Clinic Maja” in Niš, Serbia, between December 2023 and February 2026. The study was conducted in accordance with the Declaration of Helsinki and approved by the Ethics Committee of the Special Hospital for Ophthalmology “Clinic Maja” (protocol code 0/723 on 20 December 2023). All patients provided written informed consent for intravitreal treatment and for the use of anonymized clinical data for research purposes prior to therapy initiation. The cohort size was determined not by convenience but by deliberately restrictive eligibility criteria: the study was designed to isolate the treatment effect of a single uniform protocol by excluding all sources of treatment heterogeneity: prior intravitreal therapy, prior macular laser, switch or loading-only follow-up, and personalized treat-and-extend dosing. The resulting cohort of 20 eyes represents the complete population of consecutive patients meeting these stringent homogeneity criteria during the enrollment window. Because the primary objective was the precise characterization of the within-protocol treatment effect rather than testing a between-group superiority hypothesis, a conventional comparative sample-size calculation was not the appropriate design instrument; instead, the adequacy of the realized sample size was evaluated a posteriori through a pre-specified minimum detectable effect size analysis.

### 2.2. Patient Population and Eligibility Criteria

Consecutive patients presenting at the study center from the initiation of faricimab availability through the data cutoff date were eligible for inclusion if they met all of the following criteria: (1) a confirmed diagnosis of nAMD or center-involving DME requiring intravitreal treatment; (2) treatment-naïve status—no prior intravitreal injections of any anti-VEGF agent, corticosteroid, or other pharmacological agent in the study eye; (3) no prior macular laser photocoagulation in the study eye; (4) absence of concomitant ocular disease that could independently affect visual acuity or central foveal thickness, including glaucoma with advanced visual field loss, amblyopia, significant media opacity precluding reliable OCT acquisition, or any other active retinal pathology unrelated to nAMD or DME; and (5) completion of the full 24-month follow-up period with all scheduled examinations and treatment visits.

Patients were excluded if they presented with bilateral disease requiring simultaneous bilateral treatment, had intraocular surgery within six months preceding enrollment, had evidence of concurrent choroidal neovascularization of non-AMD etiology (e.g., myopic CNV, inflammatory CNV), or had systemic conditions that posed an unacceptable procedural risk. For patients with bilateral disease, only the eye with greater baseline disease severity was included to avoid statistical dependence arising from paired eyes within the same subject. Because the cohort was assembled retrospectively from ophthalmology clinical records, the type and duration of diabetes mellitus were not systematically documented for all patients and are therefore not reported.

During the enrollment window, 47 consecutive treatment-naïve eyes initiating intravitreal faricimab for nAMD or DME were assessed for eligibility. Twenty-one eyes were excluded at the eligibility stage: 8 for prior intravitreal anti-VEGF or corticosteroid therapy, 3 for prior macular laser photocoagulation, 4 for coexisting confounding ocular pathology, 2 for bilateral disease requiring simultaneous treatment, and 4 for an enrollment date that did not allow complete 24-month follow-up before the data cutoff. The remaining 26 eyes met all eligibility criteria and commenced faricimab; of these, 6 did not complete the 24-month follow-up (withdrawal of consent, relocation, or loss to follow-up) and were therefore not eligible for the 24-month analysis per inclusion criterion (5). The final analyzed cohort thus comprised 20 eyes from 20 patients—9 with nAMD and 11 with DME—all of whom completed the full 24-month follow-up with no missing data at any analysis time point. The complete patient flow is illustrated in the diagram (Supplementary Figure S0).

### 2.3. Baseline Diagnostic Evaluation

All patients underwent a comprehensive, standardized ophthalmological examination at baseline (defined as the visit at which the first intravitreal injection was administered): slit-lamp biomicroscopy of the anterior segment (Essilor SL350, Essilor Instruments, Paris, France); indirect ophthalmoscopy and contact lens fundus biomicroscopy of the posterior segment; best-corrected visual acuity (BCVA) measured with the standard Snellen chart at 6 m in decimal notation, converted to Snellen line equivalents for statistical analysis, and, for inter-study comparability, also expressed as approximate ETDRS-letter equivalents using the conventional conversion of one Snellen line ≈ 5 ETDRS letters; and spectral-domain optical coherence tomography (SD-OCT) of the macula. The use of Snellen-derived BCVA rather than direct ETDRS measurement reflects standard regional ophthalmological practice in Serbia and is acknowledged as a methodological limitation.

Macular imaging was performed using the Zeiss Cirrus HD-OCT 5000 system (Carl Zeiss Meditec AG, Jena, Germany) with the macular cube 512 × 128 acquisition protocol. The primary quantitative OCT outcome was central foveal thickness (CFT), defined as the distance from the inner limiting membrane to the retinal pigment epithelium within the central 1-mm subfield, measured in micrometers. The diagnosis of nAMD was confirmed by choroidal neovascularization with associated exudation on SD-OCT, with fluorescein angiography (FA) and/or OCT angiography (OCTA) performed as clinically indicated. Center-involving DME was diagnosed by retinal thickening and/or intraretinal cysts within the central 1-mm subfield in the setting of diabetic retinopathy.

### 2.4. Treatment Protocol

The loading phase consisted of four consecutive monthly injections (months 0, 1, 2, and 3); the maintenance phase followed a fixed every-16-week (Q16W) schedule (months 7, 11, 15, 19, and 23), corresponding to a baseline schedule of 9 injections per patient over 24 months. Beyond this fixed backbone, additional injections were administered at the treating physician’s discretion when persistent or recurrent intraretinal or subretinal fluid was identified on OCT and/or a decrease in best-corrected visual acuity relative to the previous visit was observed at a scheduled examination; the decision to administer an additional injection was made on an individual basis using combined anatomical and functional assessment. Under these criteria, 3 patients (15.0%) received one additional injection (10 total), 13 patients (65.0%) received two additional injections (11 total), and 2 patients (10.0%) received three additional injections (12 total), yielding a cohort total of 215 injections (mean 10.75 ± 0.79 per patient; median 11; range 9–12). Because most patients received at least one injection beyond the fixed Q16W backbone, the realized regimen is more accurately described as a fixed Q16W schedule supplemented by discretionary retreatment rather than a strictly fixed protocol.

All injections were performed under sterile operating room conditions using standardized aseptic technique. The injection site was prepared with 5% povidone-iodine applied to the conjunctival sac and periocular skin. Topical anesthesia was achieved with 0.5% tetracaine hydrochloride. Injections were delivered in the superotemporal quadrant, 3.5 mm from the limbus in pseudophakic eyes and 4.0 mm in phakic eyes, using a 30-gauge needle. Intraocular pressure was measured immediately after each injection. Post-injection prophylaxis consisted of tobramycin 0.3% with dexamethasone 0.1% eye drops, administered four times daily for seven days.

### 2.5. Follow-Up Schedule and Outcome Assessments

Patients were examined the day after each injection to assess for immediate post-procedural complications. During the loading phase (months 0–4), monthly follow-up coincided with each injection, and BCVA and CFT were assessed at baseline and months 1, 2, 3, and 4. During maintenance, follow-up examinations were performed at each injection visit (months 7, 11, 15, 19, 23) and at month 24. For analysis, primary time points were baseline (month 0), end of the loading phase (month 4), and end of the study (month 24). At each visit, slit-lamp biomicroscopy, BCVA, indirect ophthalmoscopy and fundus biomicroscopy, and SD-OCT with CFT measurement were performed. Safety monitoring included ocular adverse events (intraocular inflammation, endophthalmitis, retinal detachment, retinal artery occlusion, sustained intraocular pressure elevation) and systemic adverse events (cardiovascular and thromboembolic events) at every visit.

### 2.6. Outcome Measures

Primary outcomes were (1) change in CFT (µm) from baseline to month 4 and to month 24, and (2) change in BCVA (Snellen lines and ETDRS-letter equivalents) over the same intervals. Secondary outcomes included: (i) the proportion of eyes achieving BCVA gains of ≥3 lines (~15 ETDRS letters, primary responder threshold), ≥5 lines (~25 letters, MCID), and ≥6 lines (~30 letters); (ii) the proportion achieving CFT normalization (≤305 µm) and a CFT ≤ 280 µm (≤280 µm); (iii) the proportion reaching final BCVA ≥ 7 Snellen lines (~20/40 equivalent); (iv) the magnitude of additional CFT reduction and BCVA gain from month 4 to month 24 (maintenance-phase effect); (v) correlations between CFT reduction and BCVA gain at 24 months; (vi) correlations between baseline CFT and outcomes; (vii) between-group comparisons; (viii) subgroup analyses stratified by baseline CFT (<500 vs. ≥500 µm); and (ix) visual acuity category-shift analysis. Safety outcomes included the incidence and nature of any ocular or systemic adverse events over the 24-month period.

### 2.7. Statistical Analysis

All analyses were conducted in Python 3 (Python Software Foundation) using SciPy (v1.11), NumPy (v1.24), statsmodels (v0.14), and PyMC (v5.28). Continuous variables are presented as mean ± standard deviation (SD) and, where appropriate, as median and interquartile range (IQR); categorical variables are presented as absolute frequencies and percentages. Within-group changes were assessed with paired two-tailed Student’s *t*-tests, supplemented by Wilcoxon signed-rank tests as a nonparametric verification. Between-group comparisons of continuous variables used unpaired two-tailed Student’s *t*-tests; categorical comparisons used Fisher’s exact test. Normality was assessed with the Shapiro–Wilk test. Linear associations were quantified using Pearson’s r (and Spearman’s ρ for nonparametric verification). Correlation strength was interpreted conventionally as negligible (r < 0.30), weak (0.30–0.49), moderate (0.50–0.69), strong (0.70–0.89), or very strong (r ≥ 0.90).

Effect sizes for within-group changes were calculated as Cohen’s d (mean change divided by the SD of the change) and interpreted as small (0.2), medium (0.5), large (0.8), or very large (>1.0). Cohen’s d was computed independently for three intervals—baseline to month 4 (loading phase), baseline to month 24 (overall), and month 4 to month 24 (standalone maintenance-phase effect)—to characterize the temporal dynamics of treatment response. The number needed to treat (NNT) was calculated as the inverse of the absolute risk reduction for the primary responder endpoint (≥3 Snellen lines gain), using an assumed 10% background rate of spontaneous improvement in untreated neovascular retinal disease. Statistical significance was defined as a two-tailed *p*-value below 0.05, and *p*-values are reported to three decimal places (values below 0.001 are reported as *p* < 0.001).

### 2.8. Sensitivity and Bayesian Analyses

Given the cohort’s small size, a pre-specified panel of sensitivity analyses was conducted to assess the precision of point estimates and to provide formal probabilistic bounds on the conclusions, complementing the conventional frequentist framework of Section 2.7. This analytical program was designed to ensure that the study’s inferential reliability was determined by the robustness of the statistical treatment rather than by sample size alone.

First, 95% bootstrap confidence intervals (10,000 resamples with replacement, random seed 42) were computed for all primary outcome estimates—mean CFT reduction, mean BCVA gain, and corresponding Cohen’s d values—across the nAMD group, the DME group, and the combined cohort. The bootstrap procedure is robust to non-normality and is the methodologically preferred estimator of precision for small samples, where parametric Wald-type confidence intervals are known to under-cover.

Second, a linear mixed-effects model (LMM) was fitted across the three time points (baseline, month 4, month 24) for each primary outcome, with fixed effects for time, disease group (nAMD vs. DME), and the time × group interaction, and a random intercept for patient. The LMM provides a single inferential test of the time trajectory, accounts for within-subject correlation across repeated measurements, and, critically, tests whether the temporal trajectories of CFT and BCVA differ between disease groups via the time × group interaction term.

Third, Bayesian one-sample analyses with weakly informative priors were conducted for each primary outcome within each disease group using Hamiltonian Monte Carlo (No-U-Turn Sampler, 4 chains × 2000 post-warmup draws, target accept = 0.95). Priors were Normal (0, σ = 200) for the mean parameter and HalfNormal (σ = 200) for the residual standard deviation in the CFT model, and Normal (0, σ = 5)/HalfNormal (σ = 5) in the BCVA model—wide enough to permit any plausible effect direction or magnitude. Posterior distributions of the standardized effect size (Cohen’s d) and the posterior probability of a large effect, P(|d| > 0.8|data), are reported alongside 94% highest-density intervals (HDI).

Fourth, all pre-specified *p*-values reported in Section 3 (within-group time-point comparisons, between-group comparisons, and the two primary correlation tests) were corrected for false discovery rate (FDR) using the Benjamini–Hochberg procedure at α = 0.05. Both raw and FDR-adjusted *p*-values are reported, and significance is interpreted using the FDR-adjusted values for the primary conclusions.

Fifth, a minimum detectable effect-size analysis was conducted to quantify the smallest standardized effect that the realized sample sizes could reliably detect at 80% power and α = 0.05 (two-sided). This characterizes the study’s inferential resolution independent of the observed effect magnitudes. The observed variance estimates were also used to derive prospective sample-size projections for the confirmatory single-arm and two-arm studies described in Section 4.7.

All analyses were pre-specified in a written analysis plan before the database lock; no exploratory or post hoc analyses are reported as confirmatory.

## 3. Results

### 3.1. Cohort Characteristics and Treatment Exposure

Of 26 treatment-naïve eyes that met all eligibility criteria and started faricimab, 20 (9 nAMD, 11 DME) completed the full 24-month follow-up and formed the analyzed cohort; the patient flow is summarized in Supplementary Figure S1. All 20 analyzed eyes had complete CFT and BCVA records at baseline, month 4, and month 24, with no missing data at any analysis time point. Baseline characteristics were balanced between the two disease groups for both CFT (FDR-adjusted *p* = 0.775) and BCVA (FDR-adjusted *p* = 0.837), supporting an unbiased between-group comparison. A total of 215 intravitreal faricimab injections were administered across the cohort: 2 patients (10.0%) received the standard 9-injection schedule, 3 (15.0%) received 10 injections, 13 (65.0%) received 11 injections, and 2 (10.0%) received 12 injections, with a mean of 10.75 ± 0.79 (median 11; range 9–12). No ocular or systemic adverse events attributable to faricimab were recorded throughout the 24-month period. Supplementary analyses—extended responder rates, effect-size escalation, injection efficiency, and additional correlation analyses—are reported in Supplementary Tables S1–S5, and the box-plot distribution of CFT and BCVA values at each time point is provided in Supplementary Figure S4.

### 3.2. Primary Anatomical Outcomes (CFT)

Neovascular AMD (n = 9). Mean baseline CFT was 511.00 ± 91.44 µm (median 486; IQR 452–610). After the four-injection loading phase (month 4), mean CFT decreased to 342.22 ± 59.82 µm, a reduction of 168.78 ± 69.87 µm from baseline (bootstrap 95% CI 130.8–214.3 µm; paired *t*-test *p* < 0.001; FDR-adjusted *p* < 0.001; Wilcoxon *p* = 0.004; Cohen’s d = 2.42). By month 24, CFT had declined to 324.11 ± 59.23 µm (median 311; IQR 282–350), representing a total reduction of 186.89 ± 71.92 µm (35.9 ± 9.1%; 95% CI 148.1–236.0 µm; *p* < 0.001; FDR-adjusted *p* < 0.001; d = 2.60). The additional maintenance-phase CFT reduction between months 4 and 24 was 18.11 ± 9.61 µm (95% CI 12.9–24.6 µm; *p* < 0.001; FDR-adjusted *p* < 0.001; d = 1.88), confirming continued anatomical improvement beyond the loading phase. All 9 eyes (100%) achieved a > 20% reduction in CFT. CFT normalization (≤305 µm) was reached in 3 of 9 eyes (33.3%) at month 4 and in 4 of 9 (44.4%) at month 24. Individual eye-level outcomes for the nAMD group are presented in Table 1.

Diabetic macular edema (n = 11). Mean baseline CFT was 499.09 ± 61.16 µm (median 492; IQR 468–536). After loading, mean CFT decreased to 327.18 ± 59.47 µm, a reduction of 171.91 ± 66.43 µm (95% CI 136.3–210.5 µm; *p* < 0.001; FDR-adjusted *p* < 0.001; Wilcoxon *p* = 0.001; d = 2.59). At 24 months, CFT was 301.36 ± 57.60 µm (median 297; IQR 265–314), with a total reduction of 197.73 ± 65.73 µm (39.3 ± 10.5%; 95% CI 162.5–237.3 µm; *p* < 0.001; FDR-adjusted *p* < 0.001; d = 3.01). The maintenance-phase CFT reduction was 25.82 ± 17.01 µm (95% CI 16.9–35.7 µm; *p* = 0.001; FDR-adjusted *p* = 0.001; d = 1.52). All 11 eyes (100%) achieved ≥20% CFT reduction. CFT normalization was observed in 5 of 11 eyes (45.5%) at month 4 and in 8 of 11 (72.7%) at month 24. Individual eye-level outcomes for the DME group are presented in Table 2.

### 3.3. Primary Functional Outcomes (BCVA)

Neovascular AMD. Mean baseline BCVA was 3.00 ± 1.12 Snellen lines (approximately 15 ETDRS letters; ≈20/200 Snellen equivalent). After loading, BCVA improved to 6.56 ± 1.51 lines (gain of 3.56 ± 1.13 lines, ≈18 ETDRS letters; 95% CI 3.0–4.3; *p* < 0.001; FDR-adjusted *p* < 0.001; d = 3.15). At 24 months, BCVA reached 6.89 ± 1.36 lines (≈20/50 Snellen equivalent), corresponding to a total gain of 3.89 ± 0.78 lines (~19 ETDRS letters; 95% CI 3.44–4.33; *p* < 0.001; FDR-adjusted *p* < 0.001; d = 4.97). All 9 eyes (100%) achieved ≥ 3 Snellen lines (~15 ETDRS letters) at both 4 and 24 months. The additional BCVA gain from month 4 to month 24 (0.33 ± 0.71 lines, 95% CI −0.11 to 0.78) did not reach significance after FDR correction (FDR-adjusted *p* = 0.251), indicating that BCVA gains were largely consolidated by the end of the loading phase in nAMD.

Diabetic macular edema. Mean baseline BCVA was 3.09 ± 0.83 Snellen lines (≈15 ETDRS letters; ≈20/200 Snellen equivalent). After loading, BCVA improved to 6.73 ± 1.01 lines (gain 3.64 ± 0.92, ~18 letters; 95% CI 3.09–4.18; *p* < 0.001; FDR-adjusted *p* < 0.001; d = 3.93). At 24 months, BCVA reached 7.64 ± 1.12 lines (median 8; IQR 7–9; ≈20/40 Snellen equivalent), corresponding to a total gain of 4.55 ± 1.04 lines (~23 ETDRS letters; 95% CI 4.00–5.09; *p* < 0.001; FDR-adjusted *p* < 0.001; d = 4.39). The standalone maintenance-phase BCVA gain (month 4 to month 24) was 0.91 ± 0.70 lines (95% CI 0.55–1.27; *p* = 0.002; FDR-adjusted *p* = 0.002; d = 1.30), a very large effect indicating that, in DME, the maintenance phase contributed a clinically meaningful additional functional gain beyond the loading phase. All 11 eyes (100%) achieved ≥ 3 Snellen lines, and 6 of 11 (54.5%) achieved the MCID threshold of ≥5 Snellen lines (~25 letters) at 24 months.

**Figure 1:**
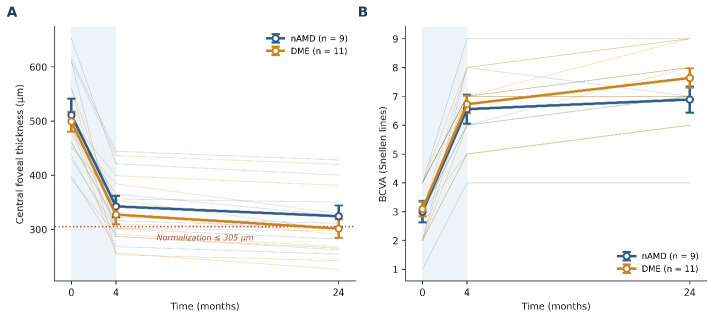
Temporal trajectories of mean central foveal thickness (CFT; **A**) and best-corrected visual acuity (BCVA; **B**) over 24 months. Individual eye trajectories are shown as faint background lines; group means ± standard error of the mean (SEM) are shown as bold curves with error bars. The shaded area denotes the loading phase (months 0–4). The dotted line in (**A**) indicates the CFT normalization threshold (≤305 µm). CFT = central foveal thickness; BCVA = best-corrected visual acuity; SEM = standard error of the mean.

The temporal trajectories of mean CFT and BCVA in both disease groups are shown in Figure 1. Both groups showed a steep, nearly parallel decline in CFT during the four-injection loading phase, followed by continued improvement throughout the fixed Q16W maintenance period; the two curves overlapped closely at every time point, visually anticipating the formal absence of a between-group difference established in Section 3.4 and Section 3.7.

### 3.4. Linear Mixed-Effects Modeling

To formally test whether the temporal trajectories of CFT and BCVA differed between the two disease groups—a question central to the dual-disease design—linear mixed-effects models were fitted across the three time points, with fixed effects for time, group, and the time × group interaction, and a random intercept per eye. For CFT, the time effect was highly significant (β = −5.57 µm per month, *p* < 0.001), whereas neither the group main effect (β = −12.55 µm, *p* = 0.70) nor the time × group interaction (β = −0.43 µm per month, *p* = 0.84) was significant. For BCVA, the time effect was again highly significant (β = +0.115 Snellen lines per month, *p* < 0.001), with no significant group main effect (*p* = 0.90) or time × group interaction (*p* = 0.51). The absence of a significant time × group interaction provides formal statistical confirmation that nAMD and DME followed parallel response trajectories under the fixed Q16W faricimab protocol—a finding consistent with the shared dual VEGF-A/Ang-2 mechanism and supporting the methodological validity of analyzing both diseases within a single cohort.

### 3.5. Bayesian Analysis with Weakly Informative Priors

Bayesian one-sample analyses with weakly informative priors and Hamiltonian Monte Carlo sampling (4 chains × 2000 draws, target acceptance rate = 0.95) yielded posterior estimates of the mean change and the standardized effect size (Cohen’s d) for each primary outcome (Table 3).

For nAMD CFT reduction at 24 months, the posterior mean was 182.7 µm (94% HDI 125.3–236.9), with posterior standardized effect size d = 2.32 (94% HDI 1.04–3.74) and posterior probability of a large effect, P(|d| > 0.8|data) = 0.99. For nAMD BCVA gain at 24 months, the posterior mean was 3.88 lines (94% HDI 3.25–4.52), with d = 4.52 (94% HDI 2.32–7.03) and P(|d| > 0.8|data) = 1.00. For DME CFT reduction at 24 months, the posterior mean was 194.8 µm (94% HDI 150.1–237.8), with d = 2.77 (94% HDI 1.43–4.20) and P(|d| > 0.8|data) > 0.99. For DME BCVA gain at 24 months, the posterior mean was 4.52 lines (94% HDI 3.80–5.21), with d = 4.07 (94% HDI 2.24–6.10) and P(|d| > 0.8|data) = 1.00.

The Bayesian framework offers a complementary inferential perspective, particularly useful with small sample sizes: even after explicitly modeling uncertainty with weakly informative priors that allow any plausible effect direction or magnitude, the posterior probability of a large clinical effect exceeded 0.99 for every primary outcome in both disease groups. This addresses, in probabilistic terms, the concern that small-sample observations might be artifacts of chance rather than reflections of a genuine underlying effect.

### 3.6. FDR-Corrected Primary Inference and Minimum Detectable Effect

All 18 pre-specified *p*-values from primary within-group, between-group, and correlation comparisons underwent Benjamini–Hochberg false-discovery-rate correction at α = 0.05. Every within-group time-point comparison for CFT remained significant after correction (all FDR-adjusted *p* ≤ 0.001), as did every within-group BCVA comparison except the maintenance-phase BCVA gain in nAMD (raw *p* = 0.195; FDR-adjusted *p* = 0.251), which we accordingly interpret as non-significant. All between-group baseline and outcome comparisons remained non-significant after correction (all FDR-adjusted *p* ≥ 0.19), confirming the absence of detectable inter-disease differences. The DME CFT–BCVA correlation (Pearson r = 0.871) remained significant after correction (FDR-adjusted *p* = 0.001); the corresponding nAMD correlation (r = 0.264, FDR-adjusted *p* = 0.59) did not, consistent with the disease-specific anatomical–functional coupling discussed in Section 4.

The minimum detectable effect-size analysis indicated that, at the realized sample sizes and 80% power (α = 0.05, two-sided), the study could reliably detect Cohen’s d ≥ 1.07 in the nAMD group, d ≥ 0.94 in the DME group, and d ≥ 0.66 in the combined cohort. Every observed primary effect size exceeded these thresholds by a wide margin (all observed d > 1.5; most > 2.5), indicating that the realized sample sizes were adequate to detect the magnitude of effect actually present and that any genuine effects of smaller magnitude would have been at risk of being missed—not the present, clearly large, effects.

### 3.7. Between-Group Comparison and Disease-Specific Anatomical–Functional Coupling

**Figure 2:**
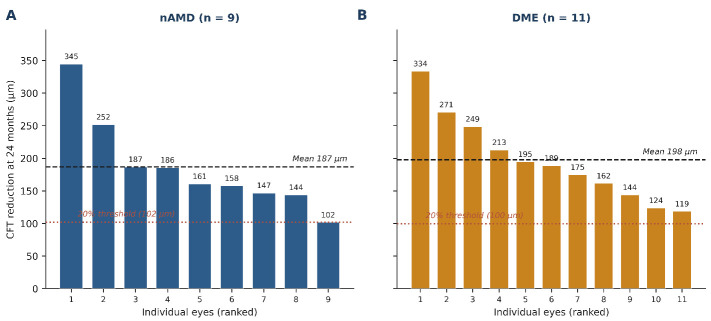
Waterfall plots of individual central foveal thickness (CFT) reductions from baseline to month 24, ranked from greatest to smallest within each disease group. (**A**) nAMD group (n = 9); (**B**) DME group (n = 11). In both panels, the dashed line indicates the group mean CFT reduction (nAMD 187 µm; DME 198 µm) and the dotted line indicates the 20% reduction threshold relative to mean baseline CFT. All bars are positive, confirming a universal anatomical response with no non-responders in either group. Bar labels denote absolute CFT reduction (µm). CFT = central foveal thickness.

The magnitude and universality of individual anatomical responses at 24 months are shown in Figure 2. Every eye in both disease groups showed a positive CFT reduction, confirming the complete absence of anatomical non-responders, while the waterfall pattern illustrates substantial inter-individual variability in response magnitude.

A comprehensive between-group comparison of baseline characteristics, primary and secondary outcomes, responder rates, and treatment exposure is presented in Table 4. No statistically significant between-group differences were observed for any primary or secondary outcome at any time point. Mean CFT reduction (nAMD 186.9 vs. DME 197.7 µm; FDR-adjusted *p* = 0.775) and mean BCVA gain (3.89 vs. 4.55 lines; FDR-adjusted *p* = 0.186) were comparable.

**Table 4:** Between-group comparison (nAMD vs. DME) of baseline characteristics, primary and secondary outcomes at 24 months, and treatment exposure.

Variable	nAMD (n = 9)	DME (n = 11)	Difference (95% CI)	*p* (Raw)	*p* (FDR)
Baseline characteristics					
CFT baseline (µm)	511.00 ± 91.44	499.09 ± 61.16	11.91 [−61.4, 79.7]	0.732	0.775
BCVA baseline (Snellen lines)	3.00 ± 1.12	3.09 ± 0.83	−0.09 [−0.99, 0.81]	0.837	0.837
Primary outcomes at 24 months					
CFT reduction (µm)	186.89 ± 71.92	197.73 ± 65.73	−10.84 [−67.1, 47.6]	0.729	0.775
CFT reduction (%)	35.9 ± 9.1	39.3 ± 10.5	−3.4 [−12.4, 5.5]	0.470	0.564
BCVA gain (Snellen lines)	3.89 ± 0.78	4.55 ± 1.04	−0.66 [−1.40, 0.09]	0.134	0.186
BCVA gain (≈ETDRS letters)	≈19.4	≈22.7	≈−3.3 [−7.0, 0.5]	0.134	0.186
Responder rates at 24 months					
CFT reduction ≥ 20%	9/9 (100%)	11/11 (100%)	–	1.000	1.000
CFT normalization (≤305 µm)	4/9 (44.4%)	8/11 (72.7%)	−28.3% [−69.6, 13.1]	0.362	0.453
CFT ≤ 280 µm	2/9 (22.2%)	4/11 (36.4%)	−14.1% [−53.0, 24.7]	0.642	0.722
BCVA gain ≥ 3 lines (~15 letters)	9/9 (100%)	11/11 (100%)	–	1.000	1.000
BCVA gain ≥ 5 lines (~25 letters, MCID)	2/9 (22.2%)	6/11 (54.5%)	−32.3% [−69.5, 4.8]	0.197	0.260
Final BCVA ≥ 7 lines (~20/40)	7/9 (77.8%)	9/11 (81.8%)	−4.0% [−41.5, 33.4]	1.000	1.000
Final BCVA ≥ 8 lines (~20/25)	2/9 (22.2%)	6/11 (54.5%)	−32.3% [−69.5, 4.8]	0.197	0.260
Treatment exposure					
Total injections (n)	11.11 ± 0.60	10.45 ± 0.82	0.66 [0.01, 1.32]	0.062	0.099
Ocular/systemic AEs	0/9 (0%)	0/11 (0%)	–	1.000	1.000

Continuous variables are presented as mean ± SD; categorical variables as n/N (%). Continuous between-group comparisons used unpaired two-tailed Student’s *t*-tests; categorical comparisons used Fisher’s exact test. Confidence intervals for between-group differences (continuous variables) are bootstrap 95% CI (10,000 resamples); for proportions, Newcombe–Wilson 95% CI. FDR-adjusted p-values were computed using the Benjamini–Hochberg procedure across all 18 pre-specified comparisons in the manuscript at α = 0.05. ETDRS-letter equivalents derived using the conventional 1 Snellen line ≈ 5 ETDRS letters conversion. CFT = central foveal thickness; BCVA = best-corrected visual acuity; MCID = minimal clinically important difference; AE = adverse event. No between-group comparison reached FDR-adjusted significance, supporting concordant response across both disease groups.

Correlations between anatomical and functional parameters at 24 months are presented in Table 5. In the DME group, CFT reduction was strongly and significantly associated with BCVA gain (Pearson r = 0.871, p < 0.001), whereas in the nAMD group this association was weak and non-significant (r = 0.264, p = 0.492), reflecting the disease-specific structural–functional coupling discussed above.

**Table 5:** Pearson and Spearman correlation coefficients between anatomical and functional parameters at 24 months.

Correlation	Group	Pearson r	Spearman ρ	*p*-Value
CFT reduction vs. BCVA gain (0 → 24 m)	nAMD	0.264	0.053	0.492
CFT reduction vs. BCVA gain (0 → 24 m)	DME	0.871	0.906	<0.001 *
Baseline CFT vs. CFT reduction at 24 m	nAMD	0.762	0.900	<0.001 *
Baseline CFT vs. CFT reduction at 24 m	DME	0.590	0.573	0.056
Baseline CFT vs. final BCVA at 24 m	nAMD	−0.769	−0.694	0.016 *
Baseline CFT vs. final BCVA at 24 m	DME	−0.232	−0.169	0.493

* *p* < 0.05. Additional correlation analyses (baseline BCVA vs. BCVA gain; maintenance-phase ΔCFT vs. final BCVA; injection count vs. outcome) are provided in Supplementary Table S3. CFT = central foveal thickness; BCVA = best-corrected visual acuity.

**Figure 3:**
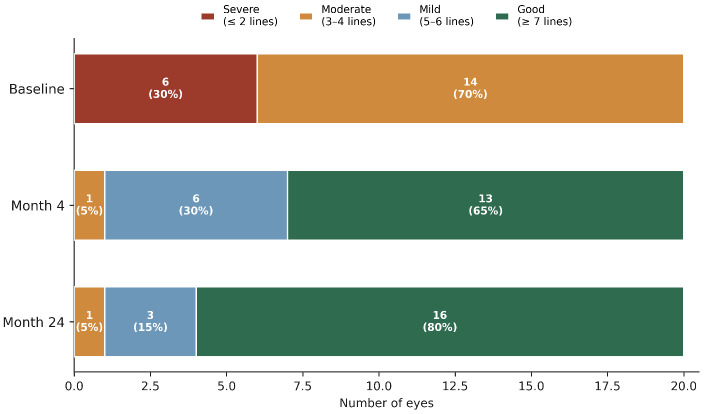
Visual acuity category distribution at baseline, month 4, and month 24 (combined cohort, n = 20). Categories: severe (≤2 Snellen lines); moderate (3–4 lines); mild (5–6 lines); good (≥7 lines). Numbers within bars indicate eye count and percentage of the total cohort.

The cohort’s distribution across visual acuity categories over the observation period is shown in Figure 3. At baseline, all 20 eyes had moderate-to-severe visual impairment, and no eye had good functional vision (≥7 Snellen lines). By month 24, 16 of 20 eyes (80%) had reached the good-vision category, and no eye remained in the severe category (≤2 lines) at any post-baseline time point.

### 3.8. Subgroup Analysis by Baseline Disease Severity

A pre-specified subgroup analysis stratified by baseline CFT severity (<500 vs. ≥500 µm) is presented in Table 6. In the nAMD group, eyes with baseline CFT ≥ 500 µm achieved significantly greater absolute CFT reductions at 24 months (261.3 vs. 149.7 µm; *p* = 0.014), whereas the corresponding trend in the DME group did not reach significance (225.2 vs. 174.8 µm; *p* = 0.223). Between-stratum differences in BCVA gain were not significant in either group. Across all four severity subgroups, all eyes achieved ≥ 3 Snellen lines at 24 months, indicating consistent functional efficacy across the full spectrum of baseline disease severity.

### 3.9. Safety

No ocular or systemic adverse events attributable to faricimab were recorded across 215 injections during the 24-month study period. Anterior segment examinations at every visit showed no signs of intraocular inflammation, sustained elevated intraocular pressure, or endophthalmitis. Posterior segment evaluation confirmed the absence of procedure-related complications, including retinal detachment, vitreous hemorrhage, or thromboembolic events.

## 4. Discussion

### 4.1. Mechanistic Context and Comparability with Phase 3 Data

Faricimab is the first bispecific antibody approved for intravitreal use, independently neutralizing both VEGF-A and angiopoietin-2 (Ang-2) [10]. The therapeutic rationale rests on accumulating evidence that VEGF-A blockade alone is mechanistically insufficient to control the multifactorial pathophysiology of nAMD and DME [7]: in nAMD, Ang-2 destabilizes the choroidal vasculature, promotes pericyte dropout, and sustains neovascular activity [8]; in DME, chronic Ang-2 upregulation exacerbates blood–retinal barrier breakdown, intensifies neuroinflammation, and drives treatment-refractory fluid accumulation [9]. Preclinical models confirm that dual Ang-2/VEGF-A inhibition provides sustained suppression of neovascular leakage beyond that achievable with VEGF-A blockade alone [16]. These mechanistic insights provide a biological foundation for the consistent and concordant effects observed across both disease groups in our cohort.

The mean CFT reductions at 24 months in our nAMD (186.9 µm, −35.9%) and DME (197.7 µm, −39.3%) cohorts fall within the range reported in the pivotal phase 3 program. TENAYA and LUCERNE demonstrated non-inferior BCVA gains and superior CST reductions for faricimab versus aflibercept at one year [11], with sustained two-year benefit and 59.0–66.9% of patients reaching Q16W dosing by week 112 [12]. YOSEMITE and RHINE for DME showed two-year mean CST reductions of 197–205 µm in the faricimab T&E arms, with >60% of patients reaching Q16W dosing at week 96 [13]. The DME CFT reduction in our cohort (197.7 µm) aligns precisely with the YOSEMITE/RHINE benchmark, providing independent real-world confirmation in a treatment-naïve South-Eastern European population that has previously been absent from international registries.

Critically, statistically significant CFT reductions persisted during the maintenance phase (months 4 to 24) in both disease groups (nAMD 18.1 µm, *p* < 0.001, d = 1.88; DME 25.8 µm, *p* = 0.001, d = 1.52), with effect sizes substantially exceeding the conventional “large” threshold (d ≥ 0.8). This indicates that fixed Q16W maintenance delivered progressive—rather than merely consolidating—anatomical benefit, consistent with phase 3 two-year data [12,13] and the mechanistic hypothesis that repeated Ang-2 blockade cumulatively stabilizes the retinal vasculature [8,16]. The Italian FARIT real-world multicenter cohort reported that at one year, 95.4% of treatment-naïve nAMD eyes and 100% of treatment-naïve DME eyes reached Q12W or longer dosing intervals (63.6% and 100%, respectively, achieving Q16W) [17], supporting Q16W as a clinically achievable maintenance interval in real-world treatment-naïve populations.

### 4.2. Interpretation of the Magnitude of Visual Acuity Gain

The BCVA gains observed in our cohort—3.89 ± 0.78 Snellen lines in nAMD (~19 ETDRS letters) and 4.55 ± 1.04 lines in DME (~23 ETDRS letters) at 24 months—are numerically higher than the +3.7 to +5.0 ETDRS letters reported in TENAYA/LUCERNE [12] and the +10.7 letters in YOSEMITE/RHINE [13]. This discrepancy reflects a fundamental difference in baseline visual acuity rather than a methodological inconsistency and is the expected statistical consequence of the well-described “ceiling/floor” phenomenon in visual acuity research. Our cohort comprised exclusively treatment-naïve patients with severe baseline visual impairment (mean BCVA 3.0–3.1 Snellen lines, approximately 20/120–20/200 Snellen equivalent; ~15 ETDRS letters), whereas registration trials enrolled patients with relatively preserved baseline acuity (73–78 ETDRS letters, approximately 20/40), leaving little headroom for substantial measured gain at the upper end of the acuity scale.

The Zarbin et al. pre-specified subgroup analysis of YOSEMITE and RHINE patients with baseline BCVA of 20/50 or worse provides direct empirical support for this interpretation [18]: in their low-baseline-acuity subgroup, faricimab produced significantly greater CST reductions than aflibercept, with comparable BCVA gains, supporting the concept that patients with more severely impaired baseline vision constitute a distinct, high-gain subpopulation with greater absolute capacity for measured functional recovery. Our cohort—drawn from a real-world public-healthcare setting where late presentation is the norm—closely resembles this 20/50-or-worse subgroup rather than the overall registration-trial population. The Wykoff et al. analysis of DRCR Network Protocol T also showed that patients with worse baseline acuity gained more letters than those with milder impairment [6]. The numerically large gains observed here should therefore be interpreted as reflecting the cohort’s baseline characteristics rather than as evidence of superior efficacy.

The 100% rate of ≥3 Snellen lines (~15 ETDRS letters) gained at 24 months is a striking finding that warrants the same baseline-anchored interpretation. With a mean baseline BCVA of ~3 Snellen lines, a ≥3-line gain corresponds to reaching the upper half of the Snellen chart—a target with substantial measurable headroom in a population starting at 20/120–20/200. We acknowledge that response rates of this magnitude are uncommon and do not claim they reflect a unique pharmacological effect. They are consistent with what would be predicted from the combination of (i) the dual-pathway mechanism of faricimab, (ii) the treatment-naïve disease state (with no prior anti-VEGF tachyphylaxis or atrophy from chronic prior therapy), (iii) the severe baseline acuity with consequent measurement headroom, and (iv) the rigorous fixed-interval dosing schedule with no missed doses over the 24-month period. We discuss further how potential biases interact with this interpretation in Section 4.6.

### 4.3. Disease-Specific Anatomical–Functional Coupling

The strong positive correlation between CFT reduction and BCVA gain in DME (r = 0.871; FDR-adjusted *p* = 0.001; Spearman ρ = 0.906) confirms that anatomical normalization was tightly coupled to functional recovery in this disease, consistent with intraretinal fluid accumulation as the dominant mechanical driver of photoreceptor dysfunction in DME. The corresponding correlation in nAMD (r = 0.264; FDR-adjusted *p* = 0.59) was non-significant, consistent with the more complex, multi-compartmental pathophysiology of choroidal neovascularization, in which irreversible outer retinal damage from chronic subretinal fluid or geographic atrophy may limit functional recovery even when anatomical normalization is achieved [19]. We acknowledge that with n = 9 in the nAMD group, the precision of the correlation estimate is low, and the result may reflect insufficient statistical power as much as a biological absence of coupling. The negative correlation between baseline CFT and final BCVA in nAMD (r = −0.769; *p* = 0.016) reinforces the well-established clinical principle that early treatment initiation, before advanced structural damage occurs, is critical for optimizing long-term functional outcomes [19].

### 4.4. Methodological Rationale for the Dual-Disease Design

A central methodological feature of this study is the simultaneous analysis of nAMD and DME within a single cohort. This approach is biologically justified by faricimab’s status as the only intravitreal agent with an identical molecular mechanism (dual VEGF-A/Ang-2 neutralization) approved for both indications, and it is supported by the linear mixed-effects model results: the absence of a time × group interaction for either CFT (*p* = 0.84) or BCVA (*p* = 0.51) provides formal statistical evidence that the two disease groups followed concordant response trajectories under identical treatment. The convergence of outcomes across biologically distinct disease entities likely reflects the shared mechanistic benefit of dual Ang-2/VEGF-A inhibition—restoring vascular stability through Tie2 signaling in both the neovascular and diabetic inflammatory contexts—rather than an intrinsic equivalence of the underlying pathologies. A scoping review of 63 real-world faricimab studies concluded that outcomes in routine practice align with registration trial data across both disease indications [14], consistent with our findings. Multiple recent real-world faricimab studies—including the Italian FARIT cohort [17], the Giancipoli et al. naïve cohort [15], and the Japanese J-CREST registry [20]—have similarly analyzed both diseases concurrently, establishing this as a methodologically accepted design in the faricimab real-world literature. A non-significant trend toward greater functional improvement in DME (BCVA gain 4.55 vs. 3.89 lines; FDR-adjusted *p* = 0.186) is biologically plausible given the tighter anatomical–functional coupling in DME and the greater proportion of DME eyes achieving CFT normalization at 24 months (72.7% vs. 44.4%), but should not be over-interpreted given the small sample.

### 4.5. Fixed Q16W Maintenance and Per-Injection Efficiency

A distinctive feature of this study is the use of a fixed Q16W maintenance schedule, supplemented by discretionary additional injections for residual or recurrent activity, rather than the personalized treat-and-extend (T&E) paradigm used in registration trials. The median number of injections in our cohort (11; range 9–12) is consistent with the median of 10–11 injections reported in the YOSEMITE/RHINE T&E arms over two years [13] and falls within the range of real-world injection frequencies across European centers [14]. The fixed protocol yielded a mean CFT reduction of 18.1 ± 6.8 µm per injection and a mean BCVA gain of 0.40 ± 0.11 Snellen lines per injection, providing a pharmacoeconomic characterization of faricimab’s per-dose efficiency under fixed-dosing conditions. The absence of a significant correlation between injection count and outcomes (all FDR-adjusted *p* > 0.05 in both groups) indicates that, within the observed range of 9–12 injections, the number of additional maintenance doses did not systematically predict response, supporting the adequacy of the standard 9-injection Q16W protocol for the majority of patients in this population. Within the limits of this small pilot cohort, these data are compatible with the possibility that a fixed-interval Q16W schedule with discretionary retreatment may be a logistically feasible option for healthcare systems where the high-frequency monitoring required for individualized T&E is resource-constrained. This hypothesis requires confirmation in adequately powered, prospective studies that directly compare fixed and personalized dosing; the present data do not establish that fixed Q16W dosing is equivalent to or preferable to T&E.

### 4.6. Limitations and Sources of Potential Bias

This study has limitations that shape the interpretation of the findings, and we discuss them in detail because they are central to the appropriate generalization of these data.

The cohort of 20 eyes, particularly the nAMD subgroup (n = 9), is a focused sample, a direct and intended consequence of the deliberately restrictive eligibility criteria that excluded all sources of treatment heterogeneity (Section 2.1). The pre-specified minimum-detectable-effect-size analysis (Section 3.6) shows that the realized sample retained 80% power to detect standardized effects of d ≈ 0.66–1.07—well below every observed primary effect (all d > 1.5)—so the principal primary outcomes were resolved with adequate statistical power. The cohort size reduces resolution for secondary analyses of smaller expected effects, most notably the nAMD anatomical–functional correlation, where a true moderate association could not be reliably distinguished from null. Point estimates for the principal outcomes are reported with bootstrap 95% confidence intervals and Bayesian credible intervals throughout, so their precision is transparently quantified rather than asserted.

The retrospective design introduces inherent sources of bias that warrant consideration, including: selection bias arising from the inclusion of consecutive but self-selected patients presenting to a single tertiary center; information bias from chart-based outcome ascertainment without masked assessors; and the absence of a contemporaneous comparator arm, which precludes causal inference and direct comparison with other anti-VEGF agents or with personalized T&E dosing under equivalent conditions. The observed 100% completion rate and absence of dropouts, while clinically favorable, may reflect selection of compliant patients with stable systemic status and supportive social circumstances and are not necessarily representative of broader real-world populations.

BCVA was measured with the Snellen chart in decimal notation and converted to Snellen line equivalents, rather than using the standardized ETDRS protocol used in registration trials. While Snellen-derived BCVA is the dominant measurement standard in routine European ophthalmological practice and was applied consistently across the cohort, it introduces measurement variability not present in ETDRS assessments and limits direct numerical comparability with phase 3 trial data. We have addressed this by reporting both Snellen and approximate ETDRS-letter equivalents throughout, but this is a methodological hedge rather than a substitute for true ETDRS measurement.

As discussed in Section 4.2, the cohort’s severe baseline visual impairment (mean BCVA ~20/120–20/200) provides substantial measurable headroom for BCVA gain compared with higher-acuity registration trial populations, contributing to the numerically large BCVA improvements observed. This is a feature of the patient population, not an artifact, but it is essential context for interpreting the magnitude of the observed effects.

The analysis does not differentiate among intraretinal fluid (IRF), subretinal fluid (SRF), and pigment epithelial detachment (PED) at each time point, limiting mechanistic interpretation of the anatomical outcomes and precluding analysis of fluid compartment-specific response patterns that have been shown to correlate with eligibility for treatment intervals and long-term durability [18,19].

While pragmatically motivated and yielding excellent outcomes, the fixed Q16W maintenance protocol does not allow assessment of whether individual patients might have benefited from extending the interval beyond Q16W or required more frequent dosing, limiting generalizability to centers employing personalized T&E regimens.

The single-center design and the limited geographic representativeness of a single Serbian ophthalmology center constrain the external validity of these findings to other Eastern and Southeastern European settings with comparable patient demographics and healthcare infrastructure.

Several variables that would inform interpretation were not available in this retrospective dataset. Systemic vascular risk factors and laboratory parameters, including blood pressure, lipid profile, glycemia, glycated hemoglobin, and inflammatory markers such as C-reactive protein, were not systematically recorded in the ophthalmology records, and the type and duration of diabetes mellitus were likewise not reliably documented; these unmeasured factors may influence treatment response and limit the depth of phenotypic characterization. In addition, per-compartment OCT fluid status (intraretinal fluid, subretinal fluid, and pigment epithelial detachment) was not graded, so anatomical thresholds such as CFT ≤ 280 µm only describe retinal thickness and do not establish a fluid-free macula. Finally, the analysis was restricted to the 20 eyes that completed follow-up; outcomes for the six patients lost to follow-up, withdrawn, or relocated before the analyzed time points were unavailable, introducing potential attrition bias whose direction cannot be determined from the available data.

### 4.7. Implications for Future Research

The focused design of this study points to clear directions for confirmatory work. Larger multicenter prospective registries with standardized ETDRS acuity measurement, systematic documentation of OCT fluid subtypes, and head-to-head comparisons of fixed Q16W versus personalized T&E protocols are needed to characterize the optimal maintenance strategy and extend these findings to broader South-Eastern European patient populations. The consistent functional benefit observed regardless of baseline BCVA severity, together with the published literature on the high-gain potential of low-baseline-acuity subgroups [6,18], supports early treatment initiation in all eligible patients, irrespective of presenting acuity, and advocates timely referral pathways in regional health systems where diagnostic delays remain a significant barrier to optimal outcomes [2]. The strong anatomical–functional coupling in DME (r = 0.871) supports CFT as a candidate surrogate endpoint for predicting functional outcomes in this disease, a finding that may inform future trial design. Finally, the injection-efficiency metrics reported here (~18 µm CFT reduction and 0.4 Snellen lines BCVA gain per injection) provide a quantitative framework for pharmacoeconomic modeling of faricimab in healthcare systems, complementing clinical outcome data from larger international registries [13,21].

A key strength of this focused cohort is that its observed effect sizes and variance estimates directly inform the rational design of the confirmatory studies it calls for. Using the observed standard deviation of CFT change (~68 µm), a future single-arm precision study aiming to estimate the mean 24-month CFT reduction to within a ±20 µm margin of error at 95% confidence would require approximately 45 eyes, or approximately 79 eyes for a tighter ±15 µm margin. A future two-arm comparative study—for example, fixed Q16W versus personalized treat-and-extend, or faricimab versus a comparator anti-VEGF agent—would require approximately 90 eyes in total (45 per arm) to detect a moderate between-protocol difference (Cohen’s d = 0.6) at 80% power and α = 0.05, or approximately 128 eyes in total to detect a smaller difference (d = 0.5). These projections, derived directly from the variance structure observed in this homogeneous cohort, provide a concrete, empirically grounded design template for the multicenter confirmatory work required to establish fixed-interval faricimab maintenance as a standard of care.

## 5. Conclusions

In this small, single-center, retrospective pilot cohort of 20 treatment-naïve eyes with nAMD or DME, a four-injection loading phase followed by a fixed Q16W maintenance schedule with discretionary retreatment was associated with durable anatomical and functional improvement over 24 months, and no ocular or systemic adverse events were recorded across 215 injections. These results are consistent with, rather than independently confirming, the efficacy of faricimab established in larger randomized programs. The high responder rates should be interpreted in light of the cohort’s severe baseline visual impairment, which provides greater measurable headroom for gain than in higher-acuity trial populations. Given the limited sample size, the single-center retrospective design, and the absence of systemic and OCT fluid-compartment data, these findings are best regarded as hypothesis-generating. They support a fixed Q16W schedule with discretionary retreatment as a logistically feasible option worth evaluating, not as an established equivalent or superior alternative to personalized treat-and-extend dosing, in adequately powered, prospective, multicenter studies using standardized ETDRS acuity assessment and systematic OCT fluid grading.

## Figures and Tables

**Table 1:** Individual and mean outcomes in the nAMD group (n = 9) across three time points.

Eye	CFT Baseline (µm)	CFT at 4 m (µm)	CFT at 24 m (µm)	CFT Red. (µm)	CFT Red. (%)	BCVA Baseline	BCVA at 4 m	BCVA at 24 m	BCVA Gain	Inj. (n)
1	460	316	302	158	34.3%	4	7	7	3	12
2	497	322	311	186	37.4%	4	7	8	4	11
3	486	365	325	161	33.1%	3	6	7	4	11
4	452	356	350	102	22.6%	3	6	7	4	10
5	429	301	282	147	34.3%	4	7	7	3	11
6	398	268	254	144	36.2%	4	9	9	5	11
7	652	421	400	252	38.7%	1	4	4	3	11
8	610	287	265	345	56.6%	2	8	7	5	12
9	615	444	428	187	30.4%	2	5	6	4	11
Mean ± SD	511.00 ± 91.44	342.22 ± 59.82	324.11 ± 59.23	186.89 ± 71.92	36.0 ± 9.1%	3.00 ± 1.12	6.56 ± 1.51	6.89 ± 1.36	3.89 ± 0.78	11.11 ± 0.60
Median [IQR]	486 [452–610]	322	311 [282–350]	–	–	3 [2–4]	7	7 [7–7]	–	11
*p*-value	–	<0.001 *	<0.001 *	–	–	–	<0.001 *	<0.001 *	–	–

* *p* < 0.001 vs. baseline (paired *t*-test and Wilcoxon signed-rank test). CFT = central foveal thickness; BCVA = best-corrected visual acuity (Snellen lines); IQR = interquartile range; Inj. = total injections administered per patient.

**Table 2:** Individual and mean outcomes in the DME group (n = 11) across three time points.

Eye	CFT Baseline (µm)	CFT at 4 m (µm)	CFT at 24 m (µm)	CFT Red. (µm)	CFT Red. (%)	BCVA Baseline	BCVA at 4 m	BCVA at 24 m	BCVA Gain	Inj. (n)
1	462	310	300	162	35.1%	3	7	7	4	11
2	392	291	268	124	31.6%	4	7	8	4	11
3	512	354	299	213	41.6%	3	7	8	5	11
4	500	399	381	119	23.8%	3	5	6	3	9
5	473	384	329	144	30.4%	4	7	7	3	11
6	492	302	297	195	39.6%	4	8	9	5	10
7	437	287	262	175	40.0%	4	7	9	5	11
8	491	253	242	249	50.7%	2	7	7	5	11
9	609	436	420	189	31.0%	2	5	6	4	11
10	560	256	226	334	59.6%	3	8	9	6	9
11	562	327	291	271	48.2%	2	6	8	6	10
Mean ± SD	499.09 ± 61.16	327.18 ± 59.47	301.36 ± 57.60	197.73 ± 65.73	39.2 ± 10.5%	3.09 ± 0.83	6.73 ± 1.01	7.64 ± 1.12	4.55 ± 1.04	10.45 ± 0.82
Median [IQR]	492 [462–560]	310	297 [262–329]	–	–	3 [2–4]	7	8 [7–9]	–	11
*p*-value	–	<0.001 *	<0.001 *	–	–	–	<0.001 *	<0.001 *	–	–

* *p* < 0.001 vs. baseline (paired *t*-test and Wilcoxon signed-rank test). CFT = central foveal thickness; BCVA = best-corrected visual acuity (Snellen lines); IQR = interquartile range; Inj. = total injections administered per patient.

**Table 3:** Bayesian posterior estimates for primary outcomes at 24 months (weakly informative priors; 4 chains × 2000 post-warmup draws; target accept = 0.95).

Outcome	Group	Posterior Mean (94% HDI)	Posterior Cohen’s d (94% HDI)	P(|d| > 0.8|Data)
CFT reduction at 24 m (µm)	nAMD (n = 9)	182.72 [125.31, 236.93]	2.32 [1.04, 3.74]	0.986
CFT reduction at 24 m (µm)	DME (n = 11)	194.77 [150.05, 237.82]	2.77 [1.43, 4.20]	0.998
BCVA gain at 24 m (Snellen lines)	nAMD (n = 9)	3.88 [3.25, 4.52]	4.52 [2.32, 7.03]	1.000
BCVA gain at 24 m (Snellen lines)	DME (n = 11)	4.52 [3.80, 5.21]	4.07 [2.24, 6.10]	1.000

CFT = central foveal thickness; BCVA = best-corrected visual acuity; HDI = highest-density interval; d = Cohen’s d (mean change divided by SD of the change). Priors: Normal (0, σ = 200) on the mean and HalfNormal (σ = 200) on the residual SD for CFT models; Normal (0, σ = 5)/HalfNormal (σ = 5) for BCVA models. P(|d| > 0.8|data) denotes the posterior probability that the standardized effect size exceeds the conventional “large” effect threshold.

**Table 6:** Subgroup analysis by baseline CFT severity (<500 µm vs. ≥500 µm).

Parameter	nAMD CFT < 500 (n = 6)	nAMD CFT ≥ 500 (n = 3)	DME CFT < 500 (n = 6)	DME CFT ≥ 500 (n = 5)	*p*-Value ^†^
CFT reduction at 24 m (µm)—Mean ± SD	149.7 ± 27.7	261.3 ± 79.4	174.8 ± 43.8	225.2 ± 81.7	0.014 */0.223
BCVA gain at 24 m (lines)—Mean ± SD	3.83 ± 0.75	4.00 ± 1.00	4.33 ± 0.82	4.80 ± 1.30	0.785/0.486
CFT ≤ 305 µm at 24 m	3/6 (50%)	1/3 (33%)	5/6 (83%)	3/5 (60%)	–
≥3 lines BCVA gain at 24 m	6/6 (100%)	3/3 (100%)	6/6 (100%)	5/5 (100%)	–

^†^ *p*-values represent independent samples *t*-test comparing CFT < 500 vs. ≥500 µm subgroups within each disease group (nAMD/DME). * *p* < 0.05. CFT = central foveal thickness; BCVA = best-corrected visual acuity.

## Data Availability

The de-identified individual patient-level data supporting the conclusions of this article will be made available by the corresponding author upon reasonable request.

## Supplementary Materials

The following supporting information can be downloaded at: https://www.mdpi.com/article/10.3390/medicina62071307/s1.
