# Supplementary material for: Real-World Faricimab for Treatment-Naïve Neovascular AMD and Diabetic Macular Edema: 24-Month Outcomes from a Single-Center Pilot Cohort in South-Eastern Europe
Source: Medicina (Kaunas). 2026 Jul 6;62(7):1307. doi: 10.3390/medicina62071307 (PMC13414216; doi:10.3390/medicina62071307)
Supplement: Supplementary file 1 [file medicina-62-01307-s001.zip › medicina-4376829-supplementary.pdf]

Supplementary Material

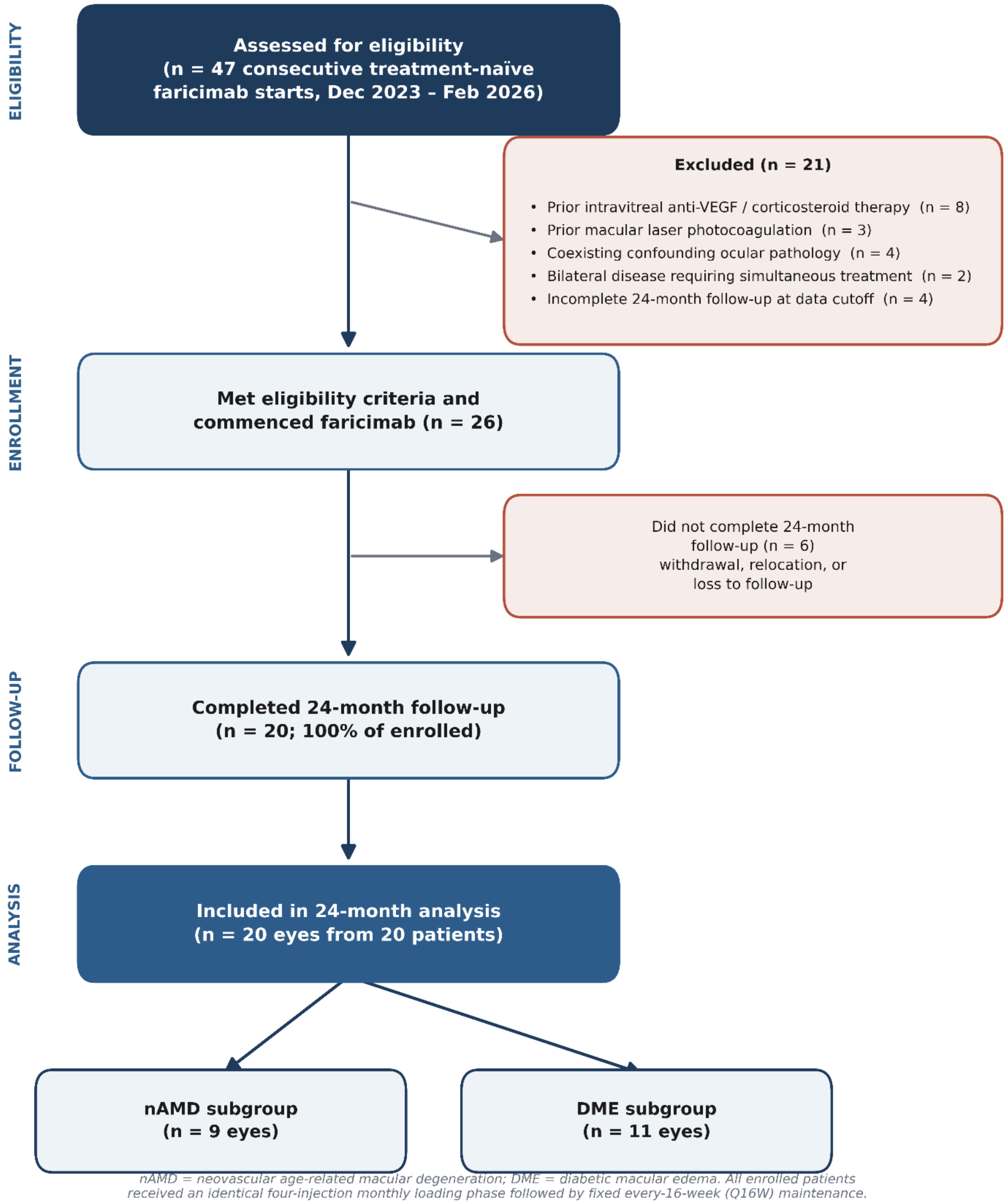

Figure S0. Flow diagram of participant inclusion and follow-up. Of 47 treatment-naïve eyes screened for eligibility, 21 were excluded at the eligibility stage and 6 of the 26 that commenced faricimab did not complete the 24-month follow-up, yielding a final analyzed cohort of 20 eyes (9 nAMD, 11 DME). Detailed exclusion criteria are listed on the diagram and in Section 3.1. DME = diabetic macular edema; nAMD = neovascular age-related macular degeneration.

Table S1. Extended responder analysis, MCID thresholds, CFT normalization rates across multiple thresholds, and number needed to treat (NNT).

Endpoint	nAMD (n = 9)	DME (n = 11)	Combined (n = 20)
≥ 3 Snellen lines gain at month 4	9/9 (100.0%)	10/11 (90.9%)	19/20 (95.0%)
≥ 3 Snellen lines gain at month 24	9/9 (100.0%)	11/11 (100.0%)	20/20 (100.0%)
MCID ≥ 5 Snellen lines gain at month 4	2/9 (22.2%)	2/11 (18.2%)	4/20 (20.0%)
MCID ≥ 5 Snellen lines gain at month 24	2/9 (22.2%)	6/11 (54.5%)	8/20 (40.0%)
≥ 6 Snellen lines gain at month 24	0/9 (0.0%)	2/11 (18.2%)	2/20 (10.0%)
Final BCVA ≥ 7 lines at month 24	7/9 (77.8%)	9/11 (81.8%)	16/20 (80.0%)
CFT ≤ 305 µm at month 4	3/9 (33.3%)	5/11 (45.5%)	8/20 (40.0%)
CFT ≤ 305 µm at month 24	4/9 (44.4%)	8/11 (72.7%)	12/20 (60.0%)
CFT ≤ 280 µm at month 4	0/9 (0.0%)	2/11 (18.2%)	2/20 (10.0%)
CFT ≤ 280 µm at month 24	2/9 (22.2%)	4/11 (36.4%)	6/20 (30.0%)
CFT ≤ 320 µm at month 24	5/9 (55.6%)	8/11 (72.7%)	13/20 (65.0%)
CFT ≤ 350 µm at month 24	7/9 (77.8%)	9/11 (81.8%)	16/20 (80.0%)
NNT for ≥ 3-line gain (vs. 10% background rate)	1.11	1.11	1.11

MCID = minimal clinically important difference (≥ 5 Snellen lines); NNT = number needed to treat, calculated as 1/(responder rate − assumed 10% background rate of spontaneous improvement in untreated neovascular retinal disease); CFT = central foveal thickness; BCVA = best-corrected visual acuity.

Table S2. Cohen's d effect sizes for CFT reduction and BCVA improvement across treatment intervals in the nAMD and DME groups.

Outcome	nAMD 0→4m	nAMD 0→24m	nAMD 4→24m	DME 0→4m	DME 0→24m	DME 4→24m	Interpretation
CFT reduction (Cohen's |d|)	**2.42**	**2.60**	**1.88**	**2.59**	**3.01**	**1.52**	Very large at all intervals in both groups
BCVA improvement (Cohen's d)	**3.15**	**4.97**	0.47	**3.93**	**4.39**	**1.30**	Overall effect escalates; DME maintenance = very large independent effect (d = 1.30)

Cohen's d conventions: 0.2 = small, 0.5 = medium, 0.8 = large, > 1.0 = very large. Absolute values reported for CFT (reduction = negative change presented as positive magnitude). CFT = central foveal thickness; BCVA = best-corrected visual acuity.

Table S3. Additional Pearson and Spearman correlation analyses: baseline BCVA vs. BCVA gain, maintenance-phase ΔCFT vs. final BCVA, and injection count vs. anatomical and functional outcomes.

Correlation	Group	Pearson r	Spearman ρ	p-value
Baseline BCVA vs. BCVA gain (0→24m)	nAMD	−0.000	−0.066	1.000
Baseline BCVA vs. BCVA gain (0→24m)	DME	−0.296	−0.280	0.377
ΔCFT (4→24m) vs. final BCVA at 24m	nAMD	−0.209	−0.422	0.590
ΔCFT (4→24m) vs. final BCVA at 24m	DME	0.101	0.169	0.767
Injection count vs. CFT reduction at 24m	nAMD	0.619	–	0.075
Injection count vs. CFT reduction at 24m	DME	−0.344	–	0.300
Injection count vs. BCVA gain at 24m	nAMD	0.030	–	0.940
Injection count vs. BCVA gain at 24m	DME	−0.203	–	0.549

None of the correlations in this table reached statistical significance (all p > 0.05), indicating that baseline visual acuity severity, continued anatomical improvement during maintenance, and number of injections received did not significantly predict the magnitude of individual treatment response. CFT = central foveal thickness; BCVA = best-corrected visual acuity; ΔCFT = CFT reduction during the maintenance phase (month 4 to month 24).

Table S4. Injection efficiency metrics: anatomical and functional gains per administered injection, and proportional contribution of loading vs. maintenance phase to total 24-month outcomes.

Metric	nAMD (n = 9)	DME (n = 11)	Combined (n = 20)
Total injections administered (n)	100	115	215
Injections per patient – Mean ± SD	11.11 ± 0.60	10.45 ± 0.82	10.75 ± 0.79
Injections per patient – Median [range]	11 [10–12]	11 [9–11]	11 [9–12]
Distribution: 9 / 10 / 11 / 12 injections	0 / 1 / 6 / 2	2 / 2 / 7 / 0	2 / 3 / 13 / 2
CFT reduction per injection (µm/inj) – Mean ± SD	16.67 ± 5.78	19.19 ± 7.52	18.06 ± 6.75
BCVA gain per injection (lines/inj) – Mean ± SD	0.351 ± 0.071	0.439 ± 0.117	0.399 ± 0.107
Loading phase contribution to total CFT reduction (%)	90.3%	86.9%	88.4%
Maintenance phase contribution to total CFT reduction (%)	9.7%	13.1%	11.6%
Loading phase contribution to total BCVA gain (%)	91.4%	80.0%	85.3%
Maintenance phase contribution to total BCVA gain (%)	8.6%	20.0%	14.7%

CFT reduction per injection = total CFT reduction at 24m divided by total injections per patient. Loading phase = months 0–4 (4 injections); maintenance phase = months 4–24 (5–8 injections depending on additional doses). CFT = central foveal thickness; BCVA = best-corrected visual acuity.

Table S5. Numerical data for the visual acuity category shift illustrated in Figure 3 of the main manuscript (combined cohort, n = 20).

BCVA Category (Snellen lines)	Baseline (n = 20)	Month 4 (n = 20)	Month 24 (n = 20)
≤ 2 lines (severe impairment)	6/20 (30.0%)	0/20 (0.0%)	0/20 (0.0%)
3–4 lines (moderate impairment)	14/20 (70.0%)	4/20 (20.0%)	1/20 (5.0%)
5–6 lines (mild impairment)	0/20 (0.0%)	10/20 (50.0%)	3/20 (15.0%)
≥ 7 lines (good functional vision)	0/20 (0.0%)	6/20 (30.0%)	16/20 (80.0%)

Category thresholds defined by Snellen line equivalents. BCVA = best-corrected visual acuity.

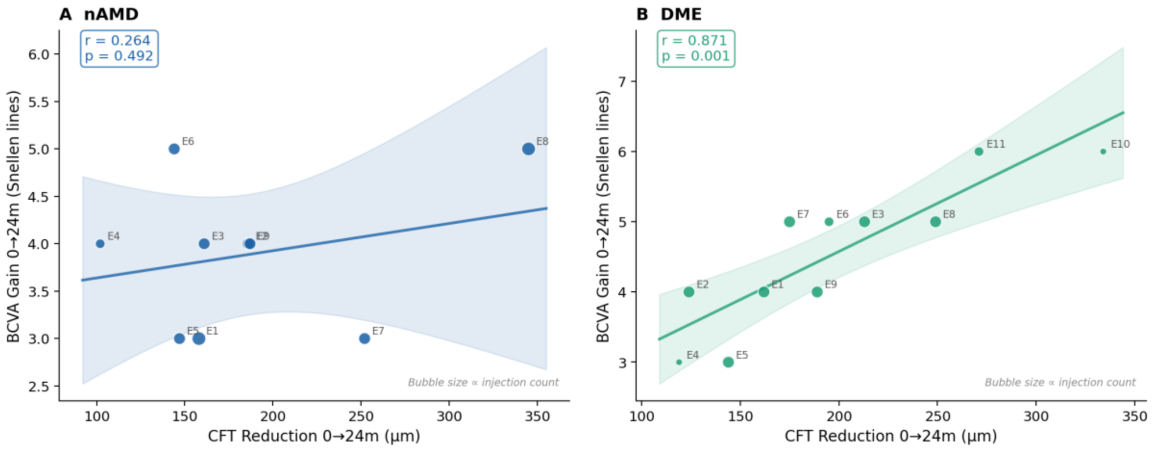

Figure S1. Scatter plots – correlation between CFT reduction and BCVA gain at 24 months with regression lines and 95% confidence interval bands. *Each data point represents one eye; bubble size is proportional to total injection count. Regression line with 95% CI band is overlaid on each panel. Panel A (nAMD): r = 0.264, p = 0.492 (not significant). Panel B (DME): r = 0.871, p < 0.001 (strong significant positive correlation). Individual eyes are labelled (E1–E9/E11). CFT = central foveal thickness; BCVA = best-corrected visual acuity; CI = confidence interval.*

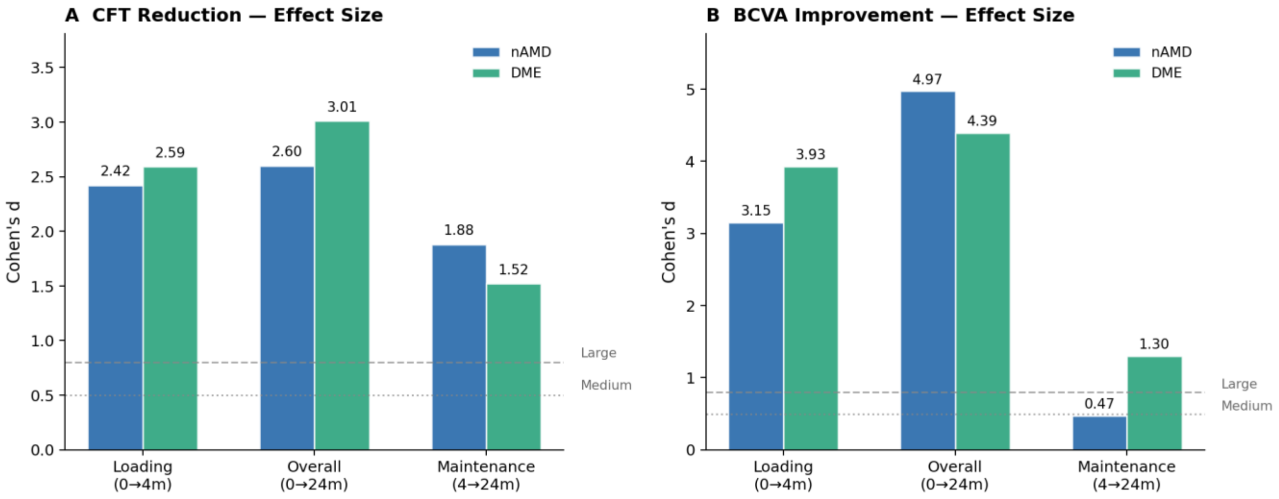

Figure S2. Cohen's d effect size escalation across treatment intervals for CFT reduction (A) and BCVA improvement (B). *Bar heights represent Cohen's d for paired within-group changes at each treatment interval. Loading = baseline to month 4; Overall = baseline to month 24; Maintenance = month 4 to month 24. Dashed line = large effect threshold (d = 0.8); dotted line = medium effect threshold (d = 0.5). The escalating effect size from loading to overall observation confirms progressive treatment benefit during Q16W maintenance. The standalone maintenance-phase BCVA effect in the DME group (d = 1.30) represents a very large independent functional contribution. CFT = central foveal thickness; BCVA = best-corrected visual acuity.*

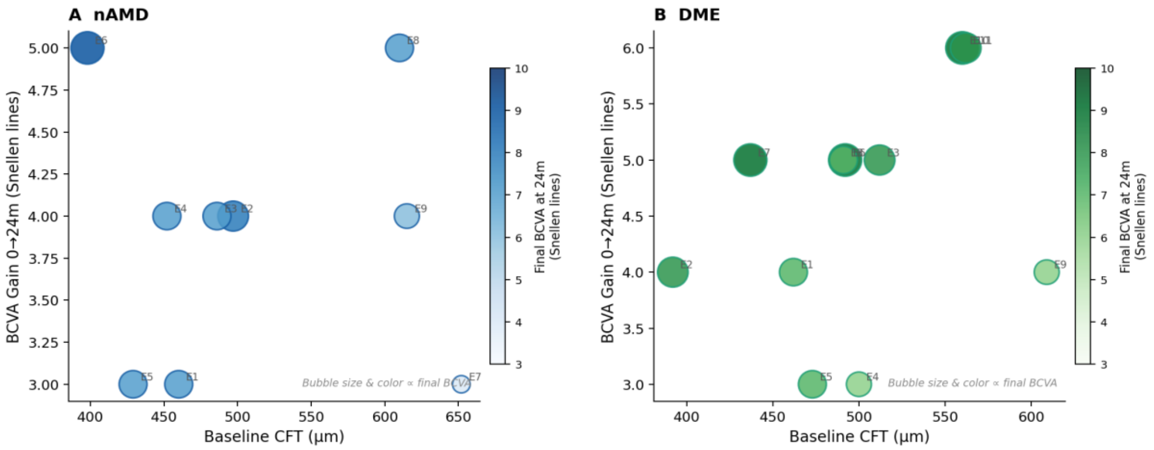

Figure S3. Bubble chart – three-dimensional outcome map: baseline CFT, BCVA gain, and final BCVA at 24 months. *x-axis = baseline CFT (μm); y-axis = BCVA gain from baseline to 24 months (Snellen lines); bubble size and color intensity ∝ final BCVA at 24 months (darker/larger = better final vision). Panel A (nAMD): considerable variability in final BCVA despite similar CFT reductions, reflecting the complex anatomical-functional relationship in neovascular AMD. Panel B (DME): eyes achieving higher final BCVA cluster toward greater CFT reductions, consistent with the strong structural-functional coupling (r = 0.871). Individual eyes labelled E1–E9/E11. CFT = central foveal thickness; BCVA = best-corrected visual acuity.*

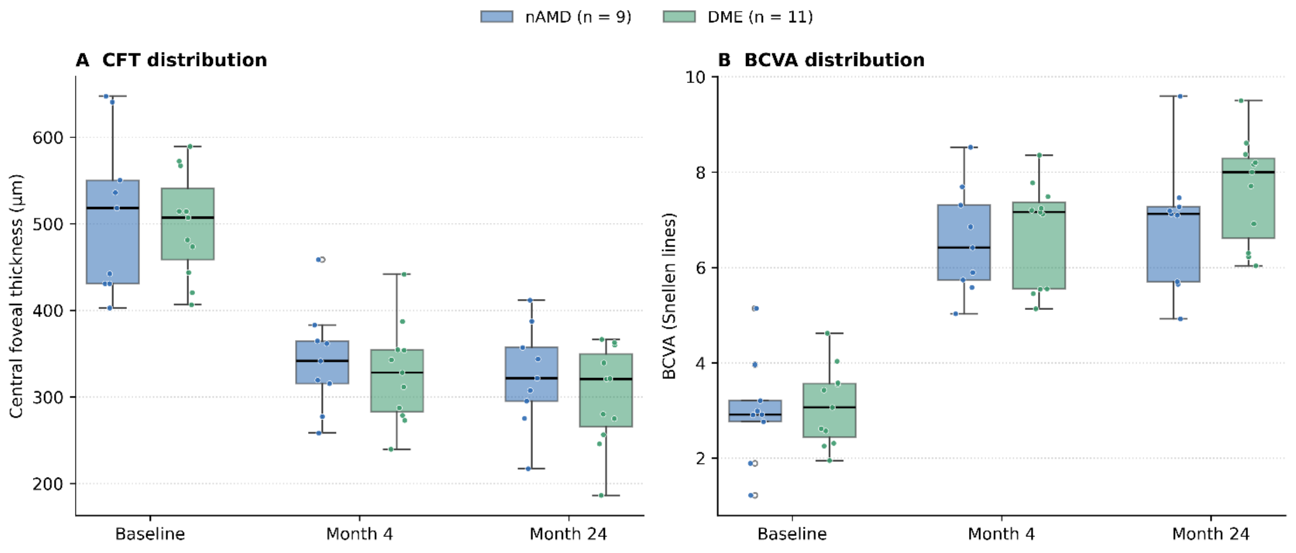

Figure S4. Box-plot distribution of central foveal thickness (A) and best-corrected visual acuity (B) at each study time point, stratified by disease group. *Boxes represent the interquartile range (IQR) with the median indicated by the horizontal line; whiskers extend to 1.5 × IQR, and individual eyes are overlaid as jittered points. Blue = nAMD (n = 9); green = DME (n = 11). Panel A shows the marked reduction in CFT from baseline to month 4, largely sustained through month 24 in both groups. Panel B shows the corresponding improvement in BCVA, with continued gain during the maintenance phase most evident in the DME group. CFT = central foveal thickness; BCVA = best-corrected visual acuity; nAMD = neovascular age-related macular degeneration; DME = diabetic macular edema; IQR = interquartile range.*
